# The Practice of Allopathy

**Published:** 1852-11

**Authors:** 


" *The Practice of Allopathy.*" — We fully concur with the editor of the New Hampshire Med. Jour., in the following remarks. Among the charges alluded to, this Journal is included, as the reader will notice by referring to the advertising sheet. The latter, however, concerns only the business department, and does not come under editorial supervision: *

" We notice in several of our exchanges an advertisement of a large drug house with this caption, and take the liberty respectfully to call the attention of editors to it. It seems apparent that to characterize the practice of medicine as *allopathy*, is to place it on the same ground as homœopathists and other *thists;* and, for ourself, we are not willing thus to be classified. We recognize but two classes among *doctors*, namely, physicians and followers of *systems*, as they are called. Claiming rank then with the former honorable body, we will not receive a title which shall confound us with the latter."

---

* Since this article was in type we have observed that the heading of the advertisement referred to is altered by direction of the advertising house.

